# Cost-effectiveness analysis of imatinib versus dasatinib in the treatment of pediatric Philadelphia chromosome-positive acute lymphoblastic leukemia when combined with conventional chemotherapy in China

**DOI:** 10.1186/s12913-023-09600-7

**Published:** 2023-06-19

**Authors:** Min Chen, Lulu Liu, Lingli Zhang, Yunzhu Lin, Xiaoxi Lu, Hao Yang, Jiaqi Ni

**Affiliations:** 1 Department of Pharmacy/Evidence-Based Pharmacy Center, West China Second University Hospital, Sichuan University, Chengdu, China; 2 Key Laboratory of Birth Defects and Related Diseases of Women and Children, Sichuan University, Ministry of Education, Chengdu, China; 3 School of Pharmaceutical Science and Technology, Tianjin University, Tianjin, China; 4 Department of Pediatric Hematology and Oncology, West China Second University Hospital, Sichuan University, Chengdu, China; 5 West China School of Pharmacy, Sichuan University, Chengdu, China

**Keywords:** Imatinib, Dasatinib, Cost-effectiveness analysis, Philadelphia chromosome-positive, Acute lymphoblastic leukemia

## Abstract

**Background:**

Tyrosine kinase inhibitors combined with conventional chemotherapy (CC) in treating Philadelphia chromosome-positive acute lymphoblastic leukemia (Ph-positive ALL) has achieved promising efficacy and safety outcomes. The study was conducted to compare the cost-effectiveness between imatinib (HANSOH Pharma, Jiangsu, China) and dasatinib (CHIATAI TIANQING Pharma, Jiangsu, China) in treating pediatric Ph-positive ALL when combined with CC from the perspective of the health system in China.

**Methods:**

A Markov model was established to simulate a hypothetical cohort of pediatric Ph-positive ALL patients receiving imatinib or dasatinib, combined with CC. The model was designed using a 10-year horizon, a 3- month cycle, and a 5% discount rate. Three health states were included: alive with progression-free survival, progressed disease, and death. Patient characteristics and transition probabilities were estimated based on clinical trials. Other relevant data, such as direct treatment costs and health utility data were extracted from published literature and Sichuan Province’s centralized procurement and supervision platform. One-way sensitivity analysis and probabilistic sensitivity analysis were performed to assess the robustness of the results. The willingness-to-pay (WTP) was set as three times China’s GDP per capita in 2021.

**Results:**

In the base-case analysis, the total medical costs were $89,701 and $101,182, and the quality-adjusted life years (QALYs) gained were 1.99 and 2.70, for imatinib and dasatinib regimens, respectively. The incremental cost-effectiveness ratio for dasatinib versus imatinib was $16,170/QALY. The probabilistic sensitivity analysis indicated that treatment with dasatinib combined with CC achieved a 96.4% probability of cost-effectiveness at a WTP threshold of $37,765/QALY.

**Conclusions:**

Dasatinib combined with CC is likely to be a cost-effective strategy compared to imatinib combination therapy for pediatric Ph-positive ALL in China at a WTP threshold of $37,765/QALY.

**Supplementary Information:**

The online version contains supplementary material available at 10.1186/s12913-023-09600-7.

## Background

Acute lymphoblastic leukemia (ALL) accounts for around 75% of all acute leukemia cases, which is the most common type of malignant neoplasm in children [1]. The 5-year survival rate in childhood ALL has greatly improved over the years and is now around 85% in China [2]. Approximately 3%- 5% of childhood ALL presents with a mutation of BCR-ABL fusion protein, which is called Philadelphia chromosome-positive ALL (Ph-positive ALL). Unlike Ph-negative ALL, these patients demonstrated rapid deterioration of disease and poor response to drug treatments, which remained challenging to manage [3].

The management of pediatric Ph-positive ALL is complicated. A number of studies have demonstrated the benefits of adding tyrosine kinase inhibitors (TKIs) early and continuously to conventional chemotherapy (CC) [4–6]. The Children’s Oncology Group (COG) trial (COG-ALL-0031) revealed that the imatinib combination therapy achieved similar clinical outcomes compared with hematopoietic stem cell transplantation (HSCT), especially in patients who had favorable responses [5]. Dasatinib has substantial clinical efficacy in treating intracranial leukemia patients, and those who failed imatinib treatment and experienced central nervous system (CNS) relapses [6]. A systematic review conducted by Chen et al. confirmed that the combination of TKIs and CC was likely to improve the event-free survival (EFS) and overall survival (OS) rates in pediatric Ph-positive ALL [7]. National Comprehensive Cancer Network (NCCN) guideline also recommended Ph-positive ALL children to be treated with chemotherapy in combination with TKIs, however, which TKI to choose was not specified [8]. The Chinese Children's Cancer Group (CCCG) trial (CCCG-ALL-2015), an open-label, phase 3 randomized controlled trial (RCT) conducted between January 1, 2015, and September 18, 2018, evaluated the efficacy and safety of oral imatinib compared with dasatinib for treating Ph-positive ALL. The results demonstrated that conventional chemotherapy combined with dasatinib showed superior outcomes compared with imatinib, and dasatinib achieved better control of CNS leukemia without the use of prophylactic cranial irradiation. Additionally, dasatinib improved the 4-year EFS and OS rates in comparison with imatinib (71.0% vs 48.9%, HR 2.36, 95% CI 1.27–4.39; and 88.4% vs 69.2%, HR 2.26, 95% CI 1.02- 5.01) [9]. Moreover, dasatinib induces hematologic and cytogenetic responses in Ph-positive ALL patients who were unable to tolerate or showed resistance to imatinib [10].

Dasatinib combination therapy seemed to be a promising first-line treatment regimen compared to imatinib for pediatric Ph-positive ALL patients, with better efficacy and comparable severe adverse event rate [7]. Nonetheless, the cost of dasatinib is much higher than imatinib referring to the government procurement and supervision platform. Cao et al. have conducted an economic analysis between imatinib and dasatinib treatment regimens for pediatric Ph-positive ALL, which revealed original dasatinib was more cost-effective compared to imatinib [11]. With China’s National Drug Pooled Procurement (NDPP) pilot program (referred to as the “4 + 7” policy in China), the price of domestic medicines dropped sharply and was far below that of imported medicines [12]. The price of the generic drug imatinib (HANSOH Pharma, Jiangsu, China 100 mg/pill) is $1.61 and dasatinib (CHIATAI TIANQING Pharma, Jiangsu, China 20 mg/pill) is $4.18, which is far lower than the original drug. Generic drug was supposed to relieve the financial burden of patient families. This study was performed to assess the cost-effectiveness of generic imatinib versus dasatinib in treating pediatric Ph-positive ALL from the perspective of health systems in China and serves as a reference for clinical decision-making.

## Methods

### Model structure

**Fig. 1:**
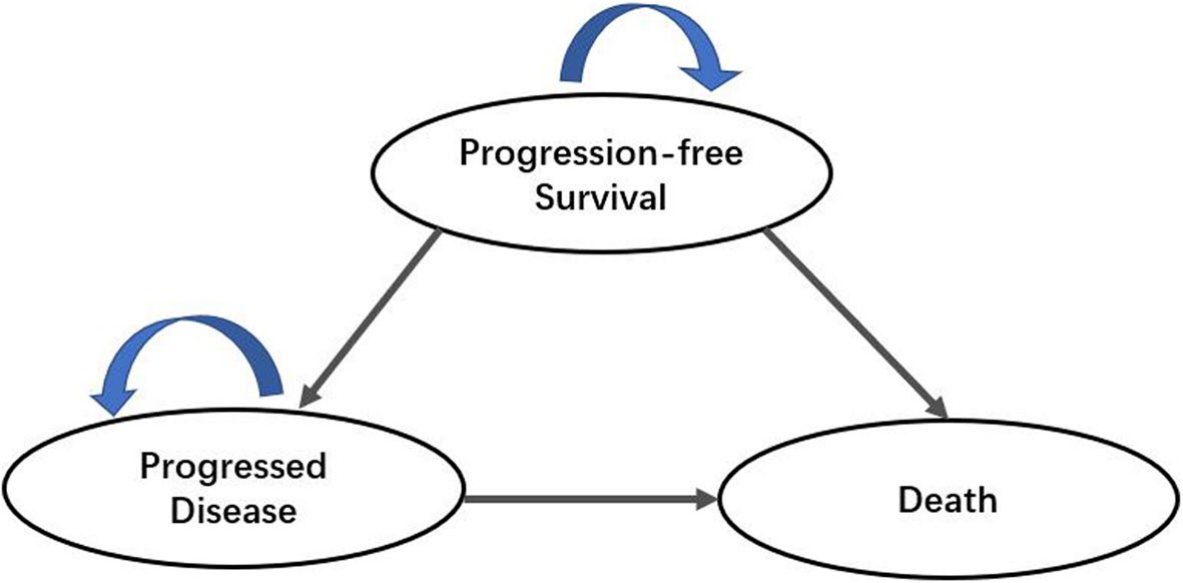
Markov model structure of the cost-effectiveness analysis

We established a Markov model with TreeAge Pro software (2017.R1.2) to evaluate the cost-effectiveness of generic imatinib compared with dasatinib in treating childhood Ph-positive ALL from the perspective of the health system. As shown in Fig. 1, three mutually exclusive health states were included: progression-free survival (PFS), progressed disease (PD), and death [13]. When the disease progressed, patients may choose a multi-drug combination of refractory chemotherapy or chimeric antigen receptor T-cell (CART) treatment to achieve complete remission and further receive HSCT. After HSCT, the patient’s status may be remission, no remission, relapse after remission, and death. In clinical practice, the probability of changing from PD state to PFS state is very low based on the expert’s opinion. Therefore, for patients simulated to experience PD, the next event would be remaining in PD state or finally death. The cycle length was 3 months and the time horizon was 10 years, including a half-cycle correction [14]. All patients were initially assumed to be PFS, with death as the terminal state. The data used in this analysis is anonymous and therefore no informed consent was needed. The reporting of this economic evaluation followed the International Society for Pharmacoeconomics and Outcomes Research (ISPOR) Consolidated Health Economic Evaluation Reporting Standards (CHEERS) checklist [15] (Supplement 1).

### Effectiveness parameters and utility estimates

The majority of inputs were obtained from published literature. Clinical experts’ advice was adopted when data was not available from the literature. In the model, data including patient characteristics and transition probabilities were extracted from clinical trials. Engauge Digitizer software was used to extract digitized data points from the EFS and OS Kaplan–Meier curves from the CCCG-ALL-2015 trial, which was a nationwide RCT conducted in pediatric patients with newly diagnosed ALL in China. Individual patient data were reconstructed using standard statistical analyses as described by Guyot et al. [16]. The following parametric survival functions were adopted: exponential, gamma, generalized gamma, gompertz, weibull, log-logistic, and log-normal. The goodness of fit was assessed with Akaike Information Criterion (AIC) and Bayesian Information Criterion (BIC) [17]. Exponential distribution was chosen based on the lowest value of AIC and BIC (Supplement 2). Time-dependent probabilities of transition at three health states were computed based on the eligible survival model.

The adverse events (AEs) data were also extracted from the literature. TKIs may cause dermatological and gastrointestinal AEs, hepatic and pancreatic disorders, musculoskeletal symptoms, fluid retention, pulmonary and cardiovascular toxicity, etc. [18]. Many of these events are temporary and resolve quickly. But some patients may experience life-changing morbidity or even death. We defined grade 3/4 events as severe adverse events (SAEs), occurring in 25% of the patients for model input [9]. Table 1 outlines a summary of the inputs and the data sources.

**Table 1:** Parameter inputs and data sources

Parameter Inputs	Mean (95%CI)	Distribution	Data Sources
**Survival model for imatinib**			
PFS^*^	AIC = 168.52, BIC = 172.19	Exponential	Estimated
OS^*^	AIC = 74.72, BIC = 82.28	Exponential	Estimated
**Survival model for dasatinib**			
PFS^*^	AIC = 103.11, BIC = 110.11	Exponential	Estimated
OS^*^	AIC = 126.55, BIC = 129.13	Exponential	Estimated
**Cost input ($)**			
Direct chemotherapy costs	27,200 (20,400–34000)	Gamma	[19, 20]
Disease progressed costs	101,047 (46,637–155,458)	Gamma	[20], expert’s opinion
Imatinib 100 mg per cycle	145 (116–174)	Gamma	Calculation
Dasatinib 20 mg per cycle	376 (301–451)	Gamma	Calculation
Management of SAEs^*^ in imatinib per cycle	891 (713–1,785)	Gamma	[21], expert’s opinion
Management of SAEs^*^ in dasatinib per cycle	1,116 (893–2,232)	Gamma	[21], expert’s opinion
**Dose of imatinib (mg/m**^**2**^**)**	300 (260–340)	Lognormal	[9, 22, 23]
**Dose of dasatinib (mg/m**^**2**^**)**	80 (40–80)	Lognormal	[9, 24]
Probability of SAEs^*^	0.25 (0.1–0.3)	Beta	[9, 13]
**Utility input**			
PFS^*^ state	0.46 (0.43–0.48)	Beta	[25]
PD^*^ state	0.21 (0.19–0.3)	Beta	[26]
**Discount rate**	0.05 (0–0.08)	Uniform	[27]

^*^PFS Progression-free survival, *OS* Overall survival, *SAEs* Severe adverse events, *PD* Progressed disease

Each health state was assigned a health utility on a scale of 0 (death) to 1 (perfect health) [28]. As studies on Ph-positive ALL health utilities were missing, and the disease presentation and prognosis of Ph-positive chronic myeloid leukemia (CML) in the accelerated or blast crisis phase behave similarly to active Ph-positive ALL, the health utilities of Ph-positive ALL were derived from studies on Ph-positive CML [14, 21]. The utility of PFS and PD states were set as 0.46 and 0.21, respectively. The utilities were then used to compute total quality-adjusted life years (QALY) for each treatment regimen. QALY is a combination of length and quality of life, with each year of life divided by the utility reflecting the quality of life.

### Cost estimates

Direct healthcare costs were calculated for chemotherapy, supportive care, outpatient clinic visits, daycare admissions, inpatient days, intensive care unit days, blood products, laboratory tests, etc. The costs were obtained from previously published studies [19, 20]. The study population and treatment regimen of these studies were comparable to CCCG-ALL-2015. In the CCCG-ALL-2015 trial, pediatric participants were treated with standard regimens as designed by CCCG, including phases of remission induction, consolidation, and continuation/reinduction therapy (Supplement 3). Asian patients tended to have higher trough concentrations compared with white patients while receiving the same dose of TKIs [29]. In the model, the imatinib daily dose was set as 300 mg/m^2^, based on CCCG-ALL2015, EsPhALL 2004, and EsPhALL 2010 studies [9, 22, 23]. Dasatinib daily dose was set as 80 mg/m^2^, based on CCCG-ALL2015 and St Jude studies [9, 24]. To calculate the per cycle dose of TKIs, we assumed that a typical patient weighed 23.6 kg and was 7.8 years old [9]. Body surface area was estimated to be 0.926m^2^ to calculate the TKI dosage. The duration of treatment was 2.5–3 years. The costs of imatinib (HANSOH pharma 100 mg/pill) and dasatinib (CHIATAI TIANQING 20 mg/pill) were obtained from the Sichuan Province Centralized Procurement and Supervision Platform (https://www.scyxzbcg.cn/). The cost of PD status was calculated with the total costs of refractory chemotherapy or CART and HSCT treatment. A total cost of $101,047 was input for PD status in the model based on published literature and opinions from clinical experts [20].

A meta-analysis performed by Fachi et al. suggested that dasatinib was more likely to cause grade 3/4 AEs compared to other TKIs [18]. In addition, dasatinib takes a higher risk of inducing grade 3/4 gastrointestinal toxicity and pleural effusion than imatinib [30]. Therefore, the cost of managing SAEs was set as 25% higher for dasatinib compared with imatinib, based on published studies [9, 13, 21]. All costs were converted to US dollars according to the average currency exchange rate in 2021 (1 $ = 6.4326 CNY, Sep.15, 2021).

### Sensitivity analysis

Deterministic and probabilistic sensitivity analyses were performed to assess the robustness of the results. In deterministic sensitivity analysis, the parameters were assigned with the lower and upper limits obtained from confidence intervals. If there is no confidence interval reported, a range of ± 20% of the base case value was adopted [31]. In addition, we conducted a one-way sensitivity analysis for all parameter inputs. Probabilistic sensitivity analysis (PSA) based on a second-order Monte Carlo simulation (1000 simulations) was performed, and cost-effectiveness acceptability curves (CEAC) were plotted. Each parameter was put into the model with different distribution types: gamma distributions were adopted for costs, whereas beta distributions were used for probabilities, proportions, and utilities [32].

The incremental cost-effectiveness ratio (ICER) was calculated as the incremental cost per QALY gained between the imatinib and dasatinib groups. The ICER threshold is described as the willingness to pay (WTP), which reflects the economic costs patients were willing to spend in order to obtain one QALY for treating the disease. Due to the lack of consensus on WTP in China, recommendations from the World Health Organization (WHO) were adopted. If ICER < gross domestic product (GDP) per capita, the increased cost was completely worthwhile, and the therapy was cost-effective; if GDP per capita < ICER < 3 times GDP per capita, the increased cost was acceptable and the therapy was cost-effective; if ICER > 3 times of GDP per capita, the added cost was not worthwhile, and the therapy was not cost-effective [27]. Therefore, the WTP value of this study was set as three times China’s GDP per capita in 2021 (GDP per capita = $12,588.3, WTP = $37,765) [33]. The discount rate was set at 5% in the model, in line with the China guidelines for pharmacoeconomic evaluations [27].

## Results

### Base-case analyses

The total cost was estimated to be $11,481 increased in dasatinib compared with imatinib, and the effectiveness was 0.71 QALYs improved in dasatinib versus imatinib. The estimated ICER for dasatinib regimen versus the imatinib regimen in the base case analysis was $16,170/ QALY, which was far below 3 times China’s GDP per capita (GDP per capita = $12,588.3) (Table 2).

**Table 2:** Base-case analyses for dasatinib and imatinib regimens

Regimens	Costs ($)	QALYs^*^	Incremental Cost ($)	Incremental QALYs^*^	ICER^*^
Imatinib combined with CC^*^	89,701	1.99	11,481	0.71	16,170
Dasatinib combined with CC^*^	101,182	2.70			

^*^CC Conventional chemotherapy, *QALYs* Quality-adjusted life years, *ICER* Incremental cost-effectiveness ratio

### Sensitivity analyses

**Fig. 2:**
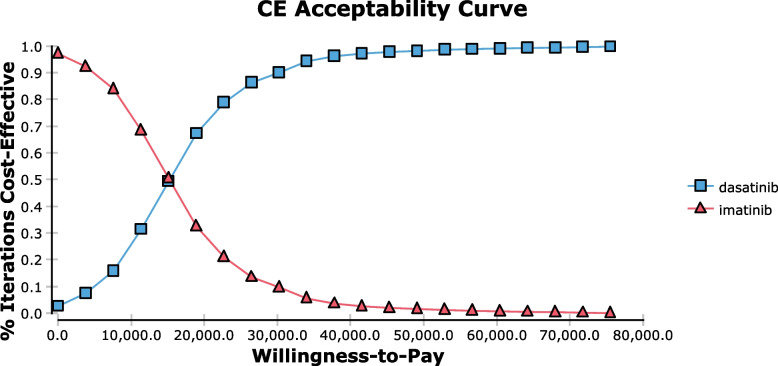
The cost-effectiveness acceptability curves for imatinib and dasatinib regimens. The vertical axes represent the probability of cost-effectiveness. The horizontal axes represent willingness-to-pay (WTP) thresholds to gain one additional quality-adjusted life year (QALY)

**Fig. 3:**
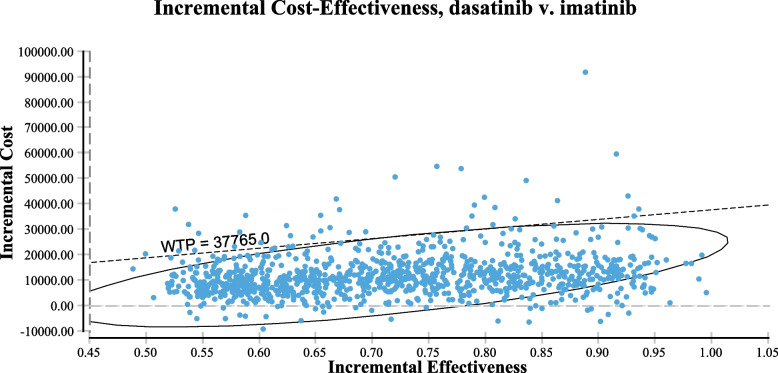
Probabilistic sensitivity analyses for imatinib versus regimens. The vertical axes represent the incremental costs. The horizontal axes represent the incremental quality-adjusted life years (QALYs) gained. The circular line demonstrated the 95%CI of incremental cost-effectiveness ratio (ICER) among the simulations, and the dotted diagonal line indicated the willingness-to-pay (WTP) threshold which had a slope of $37,765/QALY

A deterministic one-way sensitivity analysis for imatinib versus dasatinib was conducted. Parameters included price, dose, and SAEs treatment cost of TKIs, direct chemotherapy cost, the disease progressed cost, probability of SAEs, the utility of PFS and PD status, and the discount rate. Dasatinib was likely to be more cost-effective than imatinib when combined with chemotherapy, based on all of the parameters in the sensitivity analyses in a 10-year time period (Table 3). The utility value of patients in the PFS state had the greatest impact on the ICER obtained. Based on the probabilistic sensitivity analysis, the CEAC showed that dasatinib combination therapy had a 96.4% probability of being cost-effective at a WTP threshold of $37,765/QALY (Fig. 2). In the scatter plot, simulations appearing below the line favored the dasatinib combination therapy as more cost-effective (Fig. 3).

**Table 3:** One-way sensitivity analyses for dasatinib and imatinib regimens

Parameters	Base value	Low value		High value	
		Value	ICER^*^	Value	ICER^*^
Cost of imatinib 100 mg per cycle ($)	145	116	Dominates^*^	174	Dominates^*^
Cost of dasatinib 20 mg per cycle ($)	376	301	Dominates^*^	451	Dominates^*^
Dose of imatinib (mg/m^2^)	300	260	Dominates^*^	340	Dominates^*^
Dose of imatinib (mg/m^2^)	80	40	Dominates^*^	80	Dominates^*^
Cost of SAEs^*^ of imatinib ($)	891	713	Dominates^*^	1,785	Dominates^*^
Cost of SAEs^*^ of dasatinib ($)	1,116	893	Dominates^*^	2,232	Dominates^*^
Cost of direct chemotherapy ($)	27,200	20,400	Dominates^*^	34,000	Dominates^*^
Cost of disease progressed ($)	101,047	46,637	Dominates^*^	155,458	Dominates^*^
Utility of PD^*^	0.21	0.19	Dominates^*^	0.3	Dominates^*^
Utility of PFS^*^	0.46	0.43	Dominates^*^	0.48	Dominates^*^
Probability of SAEs^*^	0.25	0.1	Dominates^*^	0.3	Dominates^*^
Discount rate	0.05	0	Dominates^*^	0.08	Dominates^*^

^*^Dominates Dasatinib is more cost-effective than imatinib when combined with chemotherapy, *ICER* Incremental cost-effectiveness ratio, *SAEs* Severe adverse events, *PFS* Progression-free survival, *PD* Progressed disease

## Discussion

TKIs have demonstrated promising outcomes compared with chemotherapy alone in previous studies [4]. Cao et al. performed an economic analysis to evaluate the original drug imatinib versus dasatinib for pediatric Ph-positive ALL [11]. The set WTP threshold was 1 times per capita GDP of China in Cao’s study. Our study set the WTP threshold as 3 times per capita GDP. Both studies revealed similar results which favor dasatinib as more cost-effective compared to imatinib. There are some differences in our study. The main difference was that our study adopted a time-dependent Markov model to simulate the disease progression of pediatric Ph-positive ALL. A 3-month cycle was set other than a 1-year cycle for disease with rapid progress. In addition, the cost of managing side effects was considered, and sensitivity analysis was also performed in consideration of changes in the cost of managing side effects and dosage changes.

Dasatinib combined with CC yielded superior outcomes in treating Ph-positive ALL compared with imatinib. Additionally, dasatinib combination therapy demonstrated better control of CNS leukemia without prophylactic cranial irradiation [7, 9]. Our study revealed that, compared with imatinib, dasatinib brought an increment of 0.71 QALYs at an incremental cost of $11,481 in a 10-year time period. The results showed that dasatinib plus CC was likely to be more cost-effective compared with imatinib at WTP thresholds of $37,765 per QALY. This finding is generally robust, as shown by the results of the sensitivity analyses. In the deterministic one-way sensitivity analysis, the relationship between the ICERs and thresholds remained unchanged when lowered or upped the values of all parameters. The utility of the PFS state has a substantial impact on ICERs. The possible reason is that the PFS state occupies a larger proportion of the patient’s OS time compared with the two other states, which made it significant for ICER [31]. The daily doses of imatinib and dasatinib used in clinical trials were 260–340 mg/m^2^ and 40–80 mg/m^2^, respectively [7]. The imatinib dosage approved by Food and Drug Administration (FDA) or European Medicines Agency (EMA) for children with Ph + ALL was 340 mg/m^2^. Sensitivity analysis in our study showed that the differences in TKI dosage had no impact on the results. Probabilistic sensitivity analyses of the simultaneously various parameters illustrated that most of the scatter was below the dotted diagonal line, which indicated that dasatinib combination therapy may be more cost-effective than the imatinib combination regimen.

Studies on the WTP threshold in China were missing. Therefore, we set 3 times of GDP per capita as the WTP, according to the WHO’s standards. However, the threshold used in the medical insurance negotiation process was actually much lower than three times. Recently, Cai et al. found that the commonly used once and 3 times of GDP per capita were not necessarily empirically supported [34]. They suggested the cost-effective threshold of a QALY to be around 1.5 times of GDP per capita in China. In this scenario, the estimated ICER was also below 1.5 times of GDP per capita of China ($18,882/QALY). Likewise, the CEAC showed that dasatinib combination therapy had a 65.9% probability of being cost-effective.

Some limitations were identified in the analysis. Firstly, comparative trials for pediatric Ph-positive ALL included COG AALL0031, AALL0622, EsPhALL, and CCCG-ALL-2015 [9, 22, 23, 35]. However, only CCCG-ALL-2015 was a head-to-head randomized controlled study of imatinib and dasatinib. Clinical data were mainly extracted from CCCG-ALL-2015 in this study and the study population was Chinese patients, which limited the generalization of results. Secondly, the time horizon was set as 10 years in the model. And the survival curves extended beyond the follow-up time horizon, of which data was generated from the parametric extrapolation of survival estimates, rather than the real analysis. Well-designed RCTs with long-term follow-ups remain to be conducted to evaluate the efficacy of different TKIs. Thirdly, the costs of grade 1/2 AEs were excluded from the evaluation, which might lead to an overestimation of the economic costs. Although the sensitivity analyses showed that these variables in the model did not affect the final results. It was worth noting that the utilities were derived from a cost analysis of CML due to the absence of data pertaining to Ph-positive ALL. Therefore, research on the utility of pediatric Ph-positive ALL patients is needed in the future [11].

## Conclusions

This study demonstrated that dasatinib combined with conventional chemotherapy was likely to be a cost-effective option compared with imatinib from the perspective of the health system in China at thresholds of $37,765 per QALY. These findings will assist clinicians and the health system in optimal decision-making regarding the treatment of pediatric Ph-positive ALL.

## Supplementary Information

**Additional file 1: Supplementary Table 1.** The CHEERS 2022 checklist.

**Additional file 2.** **Supplementary Table 2.** Key parameters of survival functions. **Supplementary Table 3.** Key model parameters.

**Additional file 3.** Chinese Children Cancer Group Acute Lymphoblastic Leukemia Study: CCCG-ALL-2015 Treatment Protocol.

## Data Availability

The datasets used and/or analyzed during the current study are available from the corresponding author upon reasonable request.

## Abbreviations

CC: Conventional chemotherapy
Ph-positive ALL: Philadelphia chromosome-positive acute lymphoblastic leukemia
QALYs: Quality-adjusted life years
WTP: Willingness-to-pay
ALL: Acute lymphoblastic leukemia
TKIs: Tyrosine kinase inhibitors
COG: Children’s Oncology Group
HSCT: Hematopoietic stem cell transplantation
CNS: Central nervous system
EFS: Event-free survival
OS: Overall survival
NCCN: National Comprehensive Cancer Network
CCCG: Chinese Children's Cancer Group
RCT: Randomized controlled trial
PFS: Progression-free survival
PD: Progressed disease
ISPOR: International Society for Pharmacoeconomics and Outcomes Research
CHEERS: Consolidated Health Economic Evaluation Reporting Standards
AIC: Akaike information criterion
BIC: Bayesian information criterions
AEs: Adverse events
SAEs: Severe adverse events
CML: Chronic myeloid leukemia
CART: Chimeric antigen receptor T-cell
N/A: Not applicable
PSA: Probabilistic sensitivity analysis
CEAC: Cost effectiveness acceptability curves
ICER: Incremental cost-effectiveness ratio
WHO: World Health Organization
NDPP: National Drug Pooled Procurement
FDA: Food and Drug Administration
EMA: European Medicines Agency
GDP: Gross domestic product
