# Supplementary material for: Cost-effectiveness analysis of imatinib versus dasatinib in the treatment of pediatric Philadelphia chromosome-positive acute lymphoblastic leukemia when combined with conventional chemotherapy in China
Source: BMC Health Serv Res. 2023 Jun 19;23:652. doi: 10.1186/s12913-023-09600-7 (PMC10278346; doi:10.1186/s12913-023-09600-7)
Supplement: Supplementary file 1 — Additional file 1: Supplementary Table 1. The CHEERS 2022 checklist. [file 12913_2023_9600_MOESM1_ESM.docx]

**Supplement 1**

Supplementary Table 1. The CHEERS 2022 checklist

| Section/topic | Item No | Guidance for reporting | Reported in section |
| --- | --- | --- | --- |
| **Title** |  |  |  |
| Title | 1 | Identify the study as an economic evaluation and specify the interventions being compared. | P1 |
| **Abstract** |  |  |  |
| Abstract | 2 | Provide a structured summary that highlights context, key methods, results, and alternative analyses. | P1 |
| **Introduction** |  |  |  |
| Background and objectives | 3 | Give the context for the study, the study question, and its practical relevance for decision making in policy or practice. | P2 |
| **Methods** |  |  |  |
| Health economic analysis plan | 4 | Indicate whether a health economic analysis plan was developed and where available. | P2-3 |
| Study population | 5 | Describe characteristics of the study population (such as age range, demographics, socioeconomic, or clinical characteristics). | P2-3 |
| Setting and location | 6 | Provide relevant contextual information that may inﬂuence ﬁndings. | P2 |
| Comparators | 7 | Describe the interventions or strategies being compared and why chosen. | P2 |
| Perspective | 8 | State the perspective(s) adopted by the study and why chosen. | P2 |
| Time horizon | 9 | State the time horizon for the study and why appropriate. | P2 |
| Discount rate | 10 | Report the discount rate(s) and reason chosen. | P4-5 |
| Selection of outcomes | 11 | Describe what outcomes were used as the measure(s) of beneﬁt(s) and harm(s). | P3 |
| Measurement of outcomes | 12 | Describe how outcomes used to capture beneﬁt(s) and harm(s) were measured. | P3 |
| Valuation of outcomes | 13 | Describe the population and methods used to measure and value outcomes. | P3 |
| Measurement and valuation of resources and costs | 14 | Describe how costs were valued. | P4-5 |
| Currency, price date, and conversion | 15 | Report the dates of the estimated resource quantities and unit costs, plus the currency and year of conversion. | P3 |
| Rationale and description of model | 16 | If modelling is used, describe in detail and why used. Report if the model is publicly available and where it can be accessed. | P2-3, Figure 1 |
| Analytics and assumptions | 17 | Describe any methods for analysing or statistically transforming data, any extrapolation methods, and approaches for validating any model used. | P3 |
| Characterizing heterogeneity | 18 | Describe any methods used for estimating how the results of the study vary for subgroups. | Not Applicable |
| Characterizing distributional effects | 19 | Describe how impacts are distributed across different individuals or adjustments made to reﬂect priority populations. | P4, Table 1 |
| Characterizing uncertainty | 20 | Describe methods to characterise any sources of uncertainty in the analysis. | P5 |
| Approach to engagement with patients and others affected by the study | 21 | Describe any approaches to engage patients or service recipients, the general public, communities, or stakeholders (such as clinicians or payers) in the design of the study. | Not Applicable |
| **Results** |  |  |  |
| Study parameters | 22 | Report all analytic inputs (such as values, ranges, references) including uncertainty or distributional assumptions. | P4, Table 1 |
| Summary of main results | 23 | Report the mean values for the main categories of costs and outcomes of interest and summarise them in the most appropriate overall measure. | P5-7, Table 2, Figure 2, Figure 3 |
| Effect of uncertainty | 24 | Describe how uncertainty about analytic judgments, inputs, or projections affect ﬁndings. Report the effect of choice of discount rate and time horizon, if applicable. | P6, Table 3, Figure 3 |
| Effect of  engagement with patients and others affected by the study | 25 | Report on any difference patient/service recipient, general public, community, or stakeholder involvement made to the approach or ﬁndings of the study | Not Applicable |
| **Discussion** |  |  |  |
| Study ﬁndings, limitations, generalizability, and current knowledge | 26 | Report key ﬁndings, limitations, ethical or equity considerations not captured, and how these could affect patients, policy, or practice. | P7-8 |
| **Other relevant information** |  |  |  |
| **Source of funding** | 27 | Describe how the study was funded and any role of the funder in the identiﬁcation, design, conduct, and reporting of the analysis | P9 |
| Conﬂicts of interest | 28 | Report authors conﬂicts of interest according to journal or International Committee of Medical Journal Editors requirements. | P9 |
