# Supplementary material for: Cost-effectiveness analysis of imatinib versus dasatinib in the treatment of pediatric Philadelphia chromosome-positive acute lymphoblastic leukemia when combined with conventional chemotherapy in China
Source: BMC Health Serv Res. 2023 Jun 19;23:652. doi: 10.1186/s12913-023-09600-7 (PMC10278346; doi:10.1186/s12913-023-09600-7)
Supplement: Supplementary file 2 — Additional file 2. Supplementary Table 2. Key parameters of survival functions. Supplementary Table 3. Key model parameters. [file 12913_2023_9600_MOESM2_ESM.docx]

**Supplement 2**

Supplementary Table 2: ﻿Key parameters of survival functions

| Distribution | imatinib | | | | dasatinib | | | |
| --- | --- | --- | --- | --- | --- | --- | --- | --- |
|  | PFS | | OS | | PFS | | OS | |
|  | AIC | BIC | AIC | BIC | AIC | BIC | AIC | BIC |
| exponential | 168.52 | 172.19 | 74.72 | 82.28 | 103.11 | 110.11 | 126.55 | 129.13 |
| gamma | 171.57 | 176.72 | 84.34 | 89.39 | 109.07 | 114.12 | 128.55 | 133.70 |
| Generalized gamma | 169.25 | 176.98 | 82.38 | 84.90 | 107.59 | 110.67 | 127.39 | 135.12 |
| gompertz | 171.13 | 176.28 | 83.05 | 88.10 | 109.46 | 114.50 | 127.97 | 133.12 |
| weibullAF | 171.62 | 176.77 | 84.31 | 89.35 | 109.15 | 114.19 | 128.53 | 133.68 |
| weibullPH | 171.62 | 176.77 | 84.31 | 89.35 | 109.15 | 114.19 | 128.53 | 133.68 |
| loglogistic | 170.51 | 175.66 | 84.07 | 89.11 | 109.04 | 114.09 | 128.07 | 133.22 |
| lognormal | 169.62 | 173.67 | 82.82 | 87.86 | 107.94 | 112.98 | 126.64 | 131.79 |

Supplementary Table 3: Key model parameters

| Parameter | λ (mean, 95%Cl) |
| --- | --- |
| imatinib |  |
| Exponential model for PFS | 0.182 (0.128, 0.259) |
| Exponential model for OS | 0.103 (0.065, 0.161) |
| dasatinib |  |
| Exponential model for PFS | 0.080 (0.049,0.134) |
| Exponential model for OS | 0.049 (0.026, 0.091) |
