# Supplementary material for: Cost-effectiveness analysis of imatinib versus dasatinib in the treatment of pediatric Philadelphia chromosome-positive acute lymphoblastic leukemia when combined with conventional chemotherapy in China
Source: BMC Health Serv Res. 2023 Jun 19;23:652. doi: 10.1186/s12913-023-09600-7 (PMC10278346; doi:10.1186/s12913-023-09600-7)

**Supplement 3**

**Chinese Children Cancer Group Acute Lymphoblastic Leukemia Study: CCCG-ALL-2015 Treatment Protocol**

(From: Shen S, Chen X, Cai J, et al. Effect of dasatinib vs imatinib in the treatment of pediatric Philadelphia chromosome–positive acute lymphoblastic leukemia: a randomized clinical trial. JAMA Oncol, 2020, 6(3):358-366.)

The trial protocol included phases of remission induction, consolidation and continuation/reinduction therapy as follows:


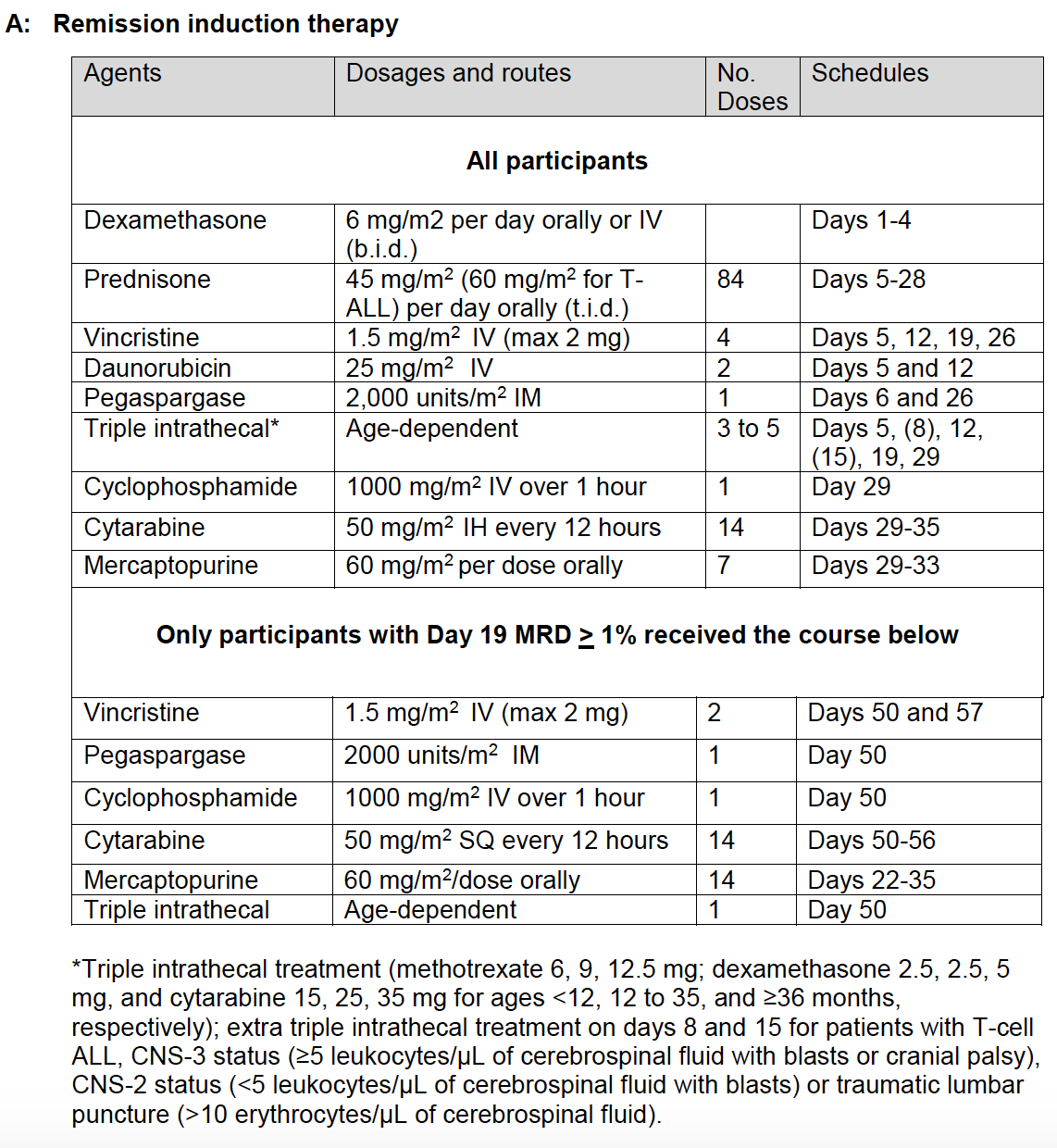


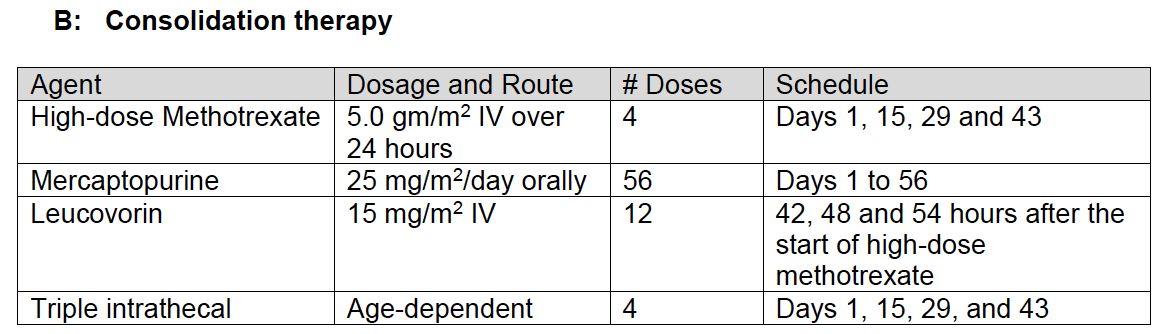


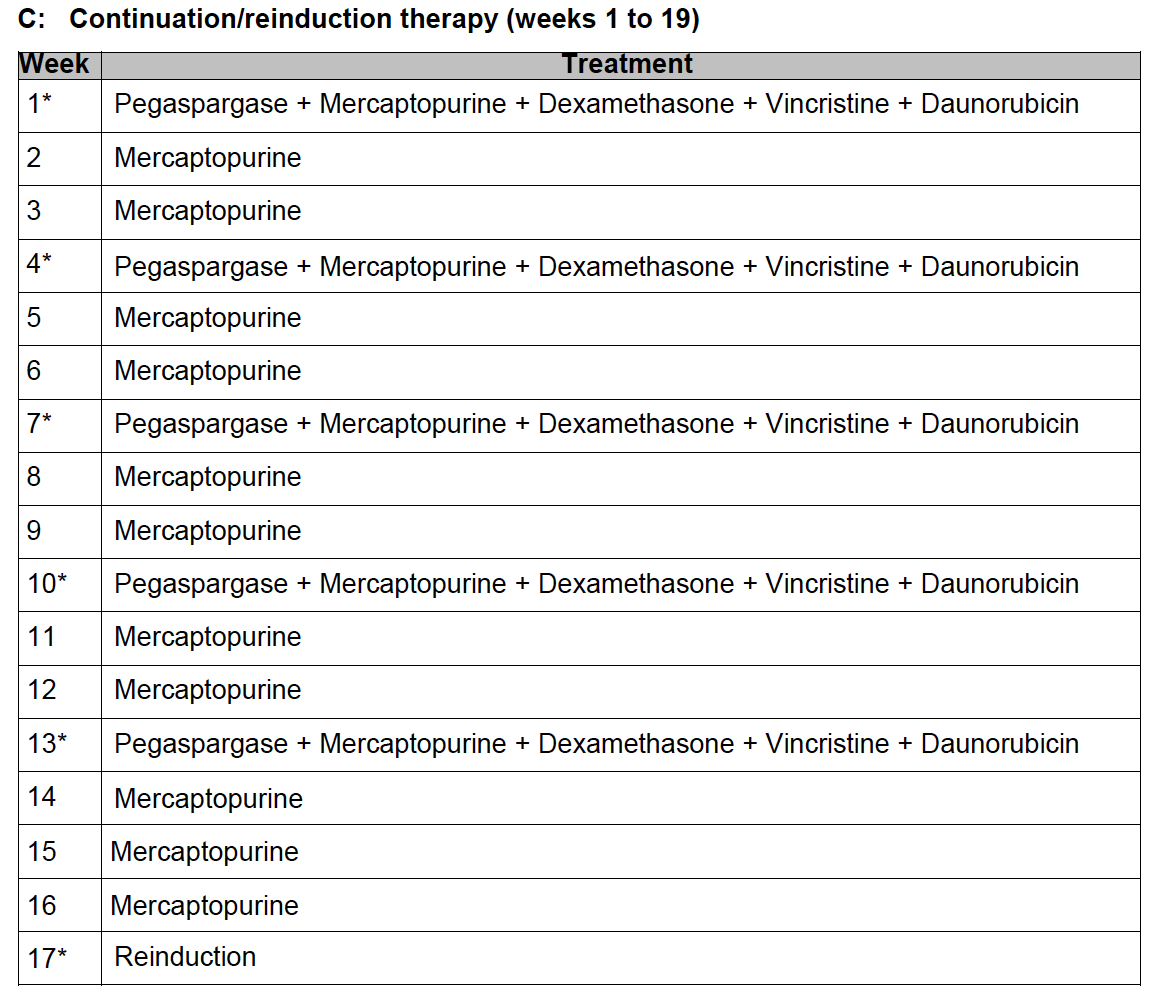


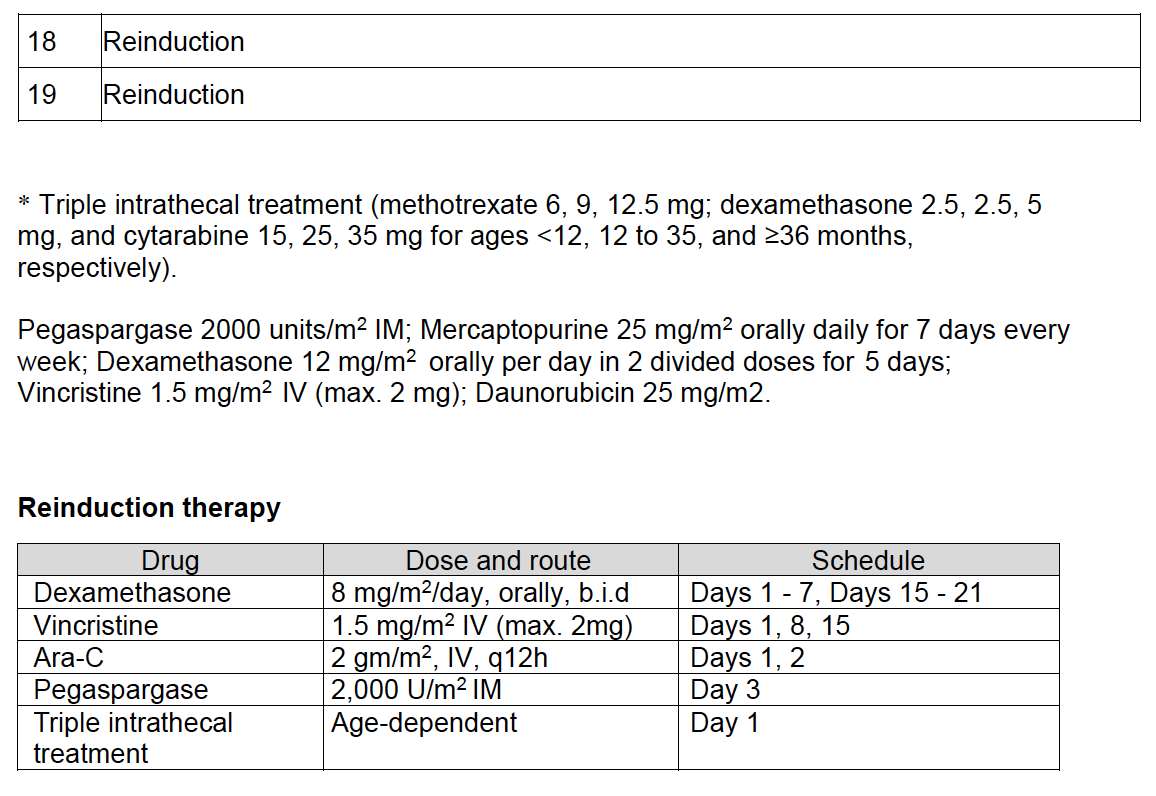


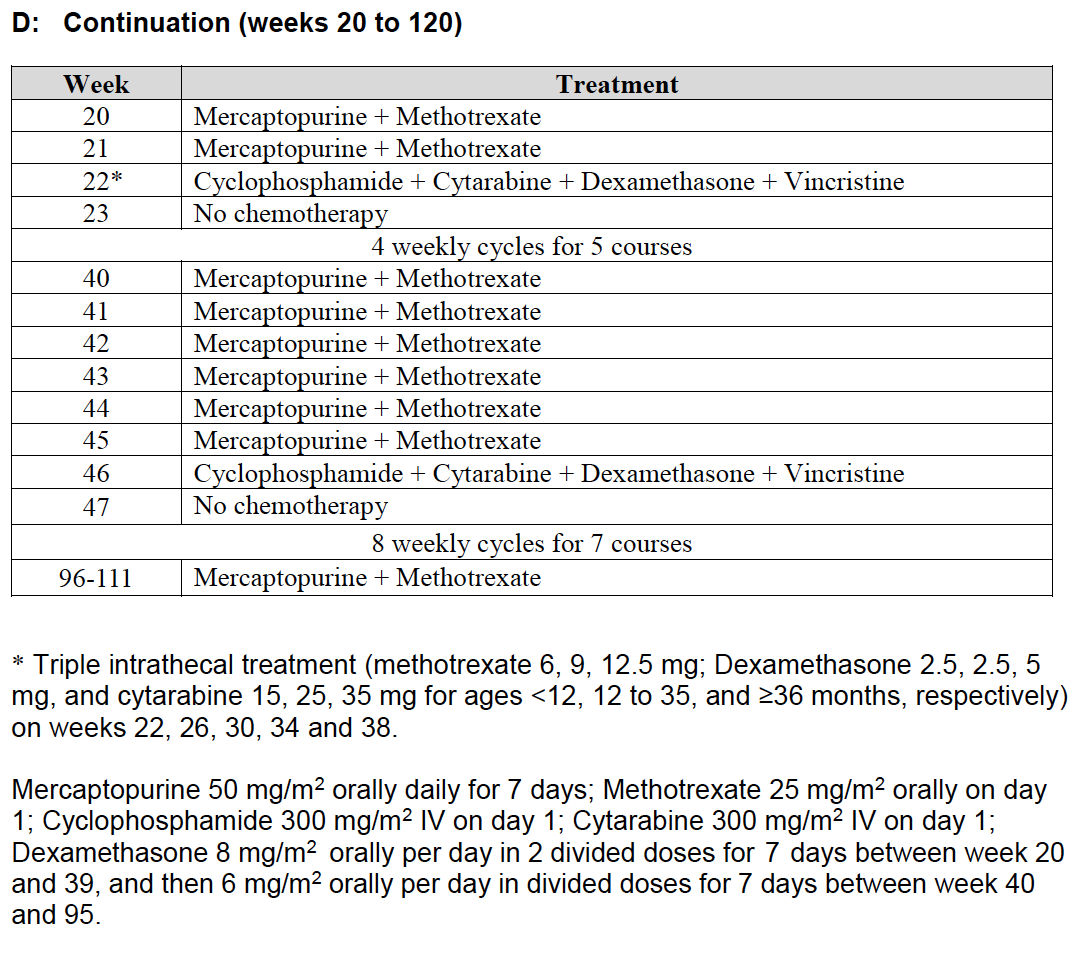

Supplement: Supplementary file 3 — Additional file 3. Chinese Children Cancer Group Acute Lymphoblastic Leukemia Study: CCCG-ALL-2015 Treatment Protocol. [file 12913_2023_9600_MOESM3_ESM.docx]
